# Uniform binding and negative catalysis at the origin of enzymes

**DOI:** 10.1002/pro.4381

**Published:** 2022-07-14

**Authors:** Elad Noor, Avi I. Flamholz, Vijay Jayaraman, Brian L. Ross, Yair Cohen, Wayne M. Patrick, Ita Gruic‐Sovulj, Dan S. Tawfik

**Affiliations:** ^1^ Department of Plant and Environmental Sciences Weizmann Institute of Science Rehovot Israel; ^2^ Division of Biology and Biological Engineering California Institute of Technology Pasadena California USA; ^3^ Resnick Sustainability Institute California Institute of Technology Pasadena CA USA; ^4^ Department of Molecular Cell Biology Weizmann Institute of Science Rehovot Israel; ^5^ Department of Biomolecular Sciences Weizmann Institute of Science Rehovot Israel; ^6^ Department of Caltech Environmental Science and Engineering California Institute of Technology Pasadena California USA; ^7^ School of Biological Sciences Victoria University of Wellington Wellington New Zealand; ^8^ Department of Chemistry, Faculty of Science University of Zagreb Zagreb Croatia

**Keywords:** enzyme evolution, negative catalysis, primordial catalyst, substrate‐handle, triose phosphate isomerase, uniform binding

## Abstract

Enzymes are well known for their catalytic abilities, some even reaching “catalytic perfection” in the sense that the reaction they catalyze has reached the physical bound of the diffusion rate. However, our growing understanding of enzyme superfamilies has revealed that only some share a catalytic chemistry while others share a substrate‐handle binding motif, for example, for a particular phosphate group. This suggests that some families emerged through a “substrate‐handle‐binding‐first” mechanism (“binding‐first” for brevity) instead of “chemistry‐first” and we are, therefore, left to wonder what the role of non‐catalytic binders might have been during enzyme evolution. In the last of their eight seminal, back‐to‐back articles from 1976, John Albery and Jeremy Knowles addressed the question of enzyme evolution by arguing that the simplest mode of enzyme evolution is what they defined as “uniform binding” (parallel stabilization of all enzyme‐bound states to the same degree). Indeed, we show that a uniform‐binding proto‐catalyst can accelerate a reaction, but only when catalysis is already present, that is, when the transition state is already stabilized to some degree. Thus, we sought an alternative explanation for the cases where substrate‐handle‐binding preceded any involvement of a catalyst. We find that evolutionary starting points that exhibit negative catalysis can redirect the reaction's course to a preferred product without need for rate acceleration or product release; that is, if they do not stabilize, or even destabilize, the transition state corresponding to an undesired product. Such a mechanism might explain the emergence of “binding‐first” enzyme families like the aldolase superfamily.

**Abbreviations**
P_1_
: desirable product selected through a binder
P_2_
: undesirable product of the dominant reaction in solution
Q: a binder which equally stabilizes S, the transition state to P_1_, and P_1_ itself (uniform binding), but stabilizes to a lesser degree, or even destabilizes, the transition state to P_2_ (negative catalysis)
S: substrate
TIM‐barrel: triose phosphate isomerase‐like barrel (an architecture that follows TPI's and yet is shared by many other enzymes)
TPI: triose phosphate isomerase
U: non‐catalytic uniform binder

## 1 PREFACE

In 2018, Dan Salah Tawfik (henceforth Danny) gave a plenary talk at the annual conference of the New Zealand Society for Biochemistry and Molecular Biology. His love of the outdoors was well known to his Kiwi friend and fellow aficionado of enzyme evolution, Wayne Patrick. And so, after the conference, an intrepid party of Australasian protein scientists—Patrick, Monica Gerth, Vic Arcus and Joel Mackay—took Danny on a two‐day hike in the mountains of New Zealand's South Island. There Wayne introduced Danny to John Albery and Jeremy Knowles' seminal 1976 article on the evolution of enzymes.^1^ Danny was particularly curious about “uniform binding,” a concept introduced in Reference 1 that involves the parallel stabilization of all enzyme‐bound states (substrate, transition state and product) to an equal degree. Indeed, Danny was sufficiently taken with the topic that he wrote a draft manuscript about uniform binding at the birth of enzyme families, which he sent to us on the evening before his fatal climbing trip.

**Figure:**
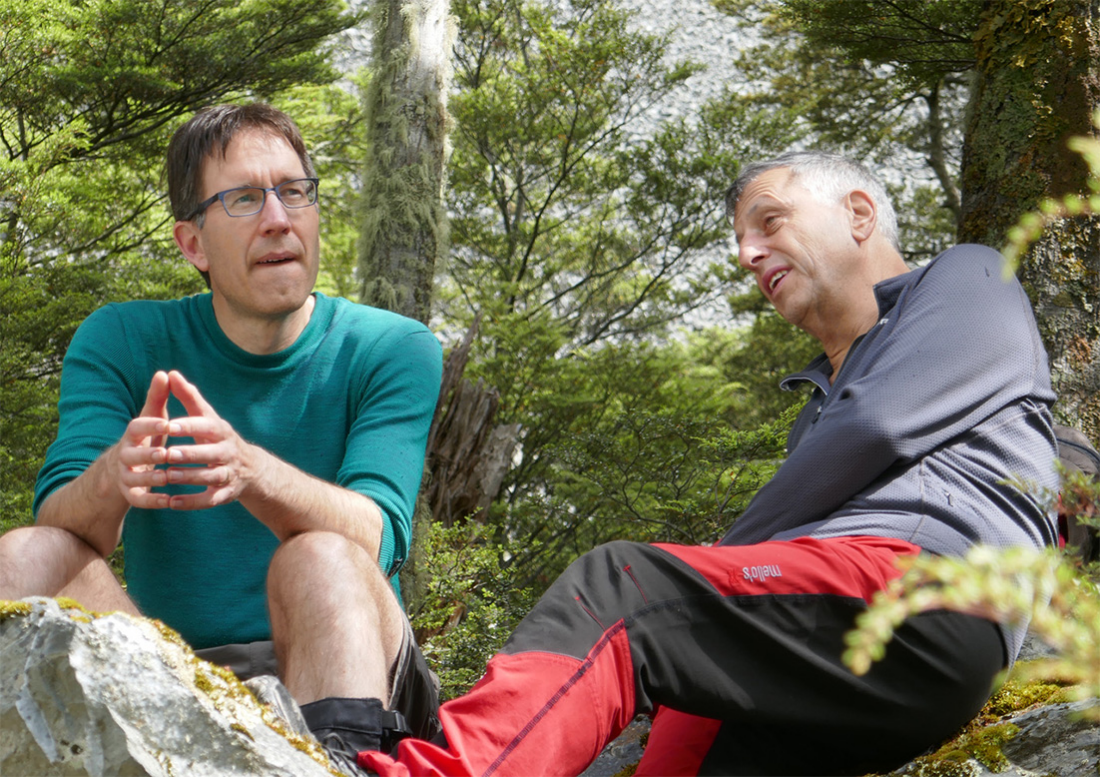
Wayne (left) and Danny (right) discussing uniform binding at Temple Stream, New Zealand (photo credit: Prof. Joel Mackay, University of Sydney)

Danny agreed with Albery and Knowles' postulate that uniform binding can arise more “easily” through mutation and selection than, for example, differential stabilization of the transition state, the latter being the textbook explanation of enzyme catalysis.^2^ Albery and Knowles showed mathematically that uniform binding can increase enzyme efficiency by several orders of magnitude, where efficiency was defined as the specific rate of substrate turnover in reference reactant concentrations.^1^ They further argued that the contribution of uniform binding to catalysis explains why common “handles,” such as phosphate, are found ubiquitously on the substrates of enzymes. Danny heartily agreed that such handles are important clues to understanding enzyme evolution, but he did not believe that uniform binding could improve catalysis, at least not in the traditional sense.

Danny's draft manuscript described a model of uniform binding where the substrate, transition state, and product of an irreversible unimolecular reaction with a single barrier are equally stabilized by binding. In addition to considering an irreversible model with only one barrier (as compared to Albery and Knowles' reversible model with three barriers), Danny's approach differed in perspective. He was focused on the early evolution of enzymes and asked whether uniform binding could provide rate acceleration relative to the uncatalyzed reaction. He, therefore, considered the total rate of conversion, that is, the sum of solution and bound rates, and correctly argued that a non‐catalytic uniform binder—that is, one that does not alter the barrier to the transition state—cannot provide rate acceleration. This motivated Danny to explore other ways in which the binding of non‐reactive substrate handles might be biologically “useful,” since, as explained below, he felt that the accumulated evidence supported the idea that several modern enzyme families arose from ancestors that recognized a specific handle (e.g., phosphate), rather than ancestors that catalyzed a particular chemistry.

It took us almost a year of combined effort to finalize Danny's manuscript in his absence, while doing our best to maintain the original scope and flow covering the evolution of enzyme superfamilies as well as models of enzyme kinetics. We were honored to wrestle with the classics in Danny's memory and wish dearly that we could discuss the topic with him over coffee, hummus or a hike, as was his custom. Danny was fond of saying that scientists should speculate louder and more frequently, that the arguments spurred by speculation make us better thinkers and that the right answers shake themselves out in the fracas. We miss Danny dearly, but our spirits are buoyed by having escorted him in one last speculation about one of his favorite topics: the early evolution of enzyme catalysis.

## 2 INTRODUCTION

How a new enzyme emerges and how evolution shapes its active site toward higher catalytic efficiency and selectivity has been a topic of great interest to biochemists and enzymologists for nearly half a century.^1^, ^3^, ^4^, ^5^ Here, we pay tribute to Albery and Knowles' contribution to this area of research, and critically reexamine the “uniform binding” concept that they introduced.

In their 1976 article, Albery and Knowles provided a conceptual and mathematical framework, an efficiency function, that compares a simple chemical catalyst (e.g., acetate) to an enzyme.^1^ Foremost, this function allows us to examine the extent to which an enzyme is optimized towards catalytic perfection (basically toward *k*_*cat*_/*K*_*M*_ matching the diffusion rate—another concept that they introduced in the same paper^1^, ^6^). They developed their model with reference to the painstaking experimental data they had collected on triose phosphate isomerase (to avoid confusion, the enzyme itself is abbreviated here as TPI while the fold named after it is dubbed the TIM‐barrel). According to their model, catalytic efficiency could be improved in three ways, each altering the reaction's free energy profile in a different manner. Two of these require developing selective mechanisms that distinguish between the substrate and product, or specifically interact with the transition state, and so the authors expected them to appear only in later stages of an enzyme's evolution.^1^ The third mechanism, uniform binding, describes cases where all enzyme‐bound states are stabilized to the same extent and has the advantage of being much easier to evolve.

Although uniform stabilization of the bound states does not increase the rate of the catalytic step—that is, the conversion from the bound substrate to the bound product—it can still increase the overall rate of substrate turnover in many scenarios, as described in References 1, 2, 6. Albery and Knowles' analysis presented a likely evolutionary path for TPI, with uniform binding improving upon simple acid/base catalysis, and subsequent improvements on the path towards “catalytic perfection” requiring selective binding. Their assumption of pre‐existing catalysis is sensible for TPI as the isomerization can be catalyzed in solution by a carboxylate base like acetate.^1^ Moreover, it is easy to imagine a prebiotic catalyst—for example, a peptide or peptide‐polynucleotide hybrid containing a carboxylate^7^, ^8^—that could both bind TPI's phosphorylated substrate and catalyze the isomerization. In addition to increasing the efficiency of pre‐existing catalysts, uniform binding can also accelerate reactions with multiple substrates (e.g., S_1_ + S_2_ → P) even in the absence of catalytic activity, as we discuss later.

In all the cases discussed above, the evolutionary starting point involves multiple molecules—a substrate and a small molecule catalyst, or multiple substrates. This prompted us to wonder whether uniform binding is also able to accelerate the simplest unimolecular reactions. To address this point, we consider an uncatalyzed unimolecular reaction where a protein (or another molecule) U uniformly binds the substrate, the transition state, and the product. As we show below (Figure 3), U is unable to accelerate such a reaction beyond the uncatalyzed rate because uniform binding does not alter the barrier to the transition state (or transition states, in the case of multi‐step reactions). We, therefore, ask: Can such a protein, or a primordial peptide for that matter, provide any evolutionarily meaningful advantage?

Another aspect addressed here is the potential role of phosphate as a binding “handle” in the emergence of the earliest enzymes and metabolites. Albery and Knowles pointed to the abundance of phosphate moieties in metabolites, where phosphate often serves as a non‐reacting handle,^1^ as is the case with TPI's substrate. In the following sections, we examine this speculation and outline evidence in support of the hypothesis that TPI and other enzymes arose from ancestors that bound phosphate handles.

### 2.1 Enzyme evolution—Chemistry or substrate‐binding‐first?

**FIGURE 1:**
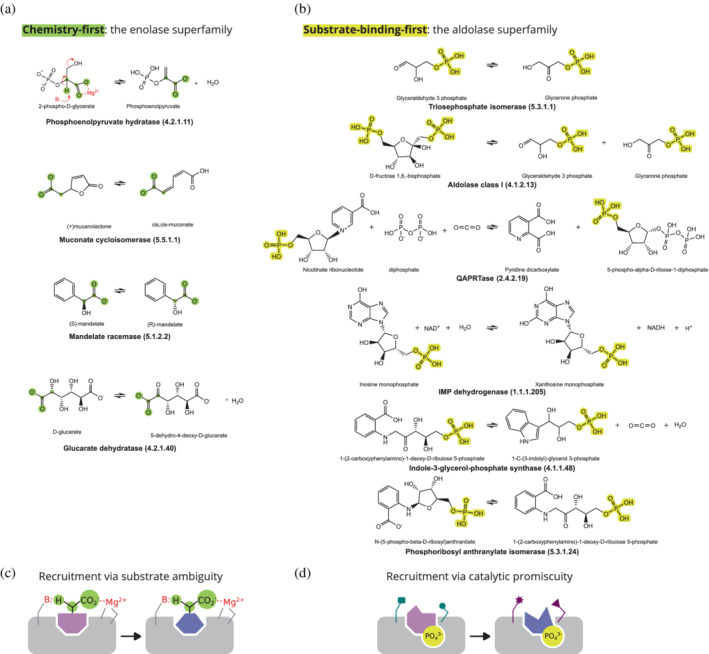
The chemistry‐first versus substrate‐handle‐binding‐first modes of emergence and the evolutionary origin of triose phosphate isomerase. (a) Emergence by chemistry‐first is demonstrated by the enolase family, members of which share a key chemical step—abstraction of a proton next to a carboxylate (shown in green). (b) In contrast, triose phosphate isomerase belongs to the aldolase superfamily, in which nearly all family members act on substrates that contain a phosphate handle—that is, a phosphate group that serves in substrate binding but is not part of the reaction. (c,d) The two modes of emergence are also reflected in the way by which new family members diverge from existing ones. (c) Chemistry‐first is manifested in recruitment via acceptance of alternative, non‐native substrates that make use of pre‐existing catalytic machinery to perform a similar reaction on the new substrate (substrate ambiguity). (d) In contrast, in catalytic promiscuity, binding of a new substrate with the same handle results in the enzyme promiscuously catalyzing a reaction that differs from the native one.

Since 1976, we have learned a great deal about enzyme evolution, foremost by examining enzyme families and superfamilies. These studies led to a model by which enzyme evolution occurs via shared chemistry and diverse substrate binding.^9^ In other words, enzyme families and superfamilies originated from a progenitor that catalyzed a key chemical step on a relatively broad range of substrates. With time, duplication and divergence gave rise to different enzymes that perform the same, or similar chemistry, each on a different substrate. The paradigm of “chemistry‐first” is well illustrated by the enolase superfamily, members of which catalyze deprotonation from a carbon adjacent to a carboxylate^10^ (Figure 1a). The enolase superfamily adopts the same overall fold as TPI, an architecture that became known as the TIM barrel.^11^ The active site architecture is highly conserved and nearly all enolase superfamily members share a lysine on one side of the active site, and a lysine or histidine on the other side. These act as base/acid, abstracting or donating a proton (e.g., in isomerization reactions). A magnesium di‐cation that ligates the carboxylate and stabilizes the carbanion intermediate is another conserved feature. Members of the enolase superfamily act primarily as dehydratases that form a double bond, or as isomerases (EC classes 4 and 5).

Other superfamilies that share the TIM‐barrel fold also obey the chemistry‐first paradigm, for example, amidohydrolases, which catalyze a broad range of hydrolytic reactions and share a catalytic metal center.^12^ However, as far as we can infer, nearly 4 billion years after these enzymes emerged, and despite the common overall architecture, TPI is part of another evolutionary lineage and is unrelated to the enolase or amidohydrolase superfamily—the so‐called aldolase superfamily.^13^, ^14^ In contrast to the enolase superfamily, TPI's evolutionary homologs share the binding of a common “handle” on the substrate, which usually does not take part in the reaction and hence typically remains unchanged in both the substrate and the product (though phosphate binding induces a conformational change in TPI that is involved in catalysis^15^, ^16^, ^17^). Accordingly, the aldolase superfamily members share a common phosphate‐binding motif as elaborated below. The catalyzed chemistry is, on the other hand, highly diverse, ranging from isomerases to kinases and oxidoreductases^14^ (Figure 1b).

Although our present understanding suggests that the majority of enzyme superfamilies abide by the chemistry‐first paradigm,^18^, ^19^ we do know of a few that evolved by binding a non‐reactive substrate‐handle first. Beyond the aldolase superfamily to which TPI belongs, the most notable example is the Rossmann lineage, which encompasses more than 300 families, mostly enzyme families, that share a common origin.^20^, ^21^ The Rossmann fold emerged as a nucleotide binding domain, and its most conserved functional elements mediate substrate binding, not chemistry. Specifically, binding of the nucleotide's phosphate and ribose groups (e.g., NAD^+^/NADH, which commonly serves as a co‐substrate) appears to have been present in its early ancestors.^21^ In fact, a polypeptide that comprises an element carrying both these binding motifs has been designated as the earliest precursor.^20^ The enzymatic chemistry (e.g., NAD(P)H mediated oxidation/reduction) is not only variable but also occurs at parts of the protein that emerged later.

How does each type of enzyme superfamily arise? Consider the recruitment of a new enzyme from an initial repertoire of preexisting enzymes. The starting point could be (a) an enzyme that accepts a new substrate and transforms it using its native reaction chemistry (Figure 1c); or (b) an enzyme that not only binds a new substrate differing from its native one but also promiscuously catalyzes a new reaction (Figure 1d). Laboratory evolution experiments and analyses of enzyme superfamilies teach us that the first recruitment scenario (dubbed substrate promiscuity or ambiguity) is the more likely one. Indeed, lab evolution of contemporary enzymes belonging to the aldolase superfamily (whose common origin was probably based on binding a non‐reacting phosphate group) showed that recruitment by substrate ambiguity was possible.^22^ However, even in this case, a shared substrate moiety was a key factor in enabling an enzyme to perform the same chemistry on a different substrate: the substrates of HisA and TrpF share a phosphoribosyl group. The second scenario, dubbed catalytic promiscuity, is readily observed as well, albeit less frequently than substrate ambiguity.^23^ Nevertheless, if the substrate possesses an abundant moiety, the likelihood of catalytic promiscuity greatly increases. Specifically, attachment of a phosphate moiety as a substrate handle was shown to greatly increase the likelihood of recruiting enzymes by catalytic promiscuity.

To summarize, a combination of laboratory evolution experiments and the natural history of certain large enzyme superfamilies (including the one to which Albery and Knowles' TPI belongs) indicate that recruitment via substrate binding is a feasible scenario, especially for substrates containing a phosphate handle.

### 2.2 Phosphate binding is a primordial protein function

Phosphate is arguably the most common moiety in natural metabolites.^24^ Accordingly, proteins that bind ligands with phosphate groups (phospho‐ligands) are highly abundant.^25^ Abundance relates to two factors. First, the most diverse and abundant enzyme superfamilies such as Rossmann enzymes and P‐loop NTPases are phospho‐ligand binders. In fact, more than one fifth of structures in the Protein Data Bank (PDB^26^) have Rossmann‐like domains.^27^ Second, in addition to these large superfamilies, phosphate binding has independently emerged many times. More than 250 such independent emergences could be detected in proteins that bind small metabolites containing phosphate groups (not including nucleic acids).^7^

Across evolutionary time, phosphate binding emerged in ~15% of all known protein lineages, namely in protein families or superfamilies that emerged independently of one another.^7^ In some of these lineages, phosphate binding has emerged sporadically, in a few of the many proteins that diverged along the lineage. However, in quite a few lineages, phosphate binding was the founding function. This is most evident in the ancient enzyme lineages thought to be present in the last universal common ancestor, LUCA.^28^ Notably, in 23% of these LUCA enzyme reactions (92 out of 404, see Table S1), there is a non‐reacting phosphate group that might have served as a handle. Substrate‐handle binding might have been the ancestral function of the Rossmann and ferredoxin folds, as well as some superfamilies that adopted the TIM‐barrel fold including the aldolase superfamily (Figure 1b). In other lineages, most notably in the P‐loop NTPases^20^ and HUP lineages,^29^ phosphate is part of the chemistry. Other examples of substrate‐binding‐first families include the HAD^30^ and ribonuclease H^31^, ^32^ lineages. It, therefore, seems that substrate binding was a common founding function at the earliest stages of enzyme evolution and specifically of substrates with phosphate as the binding handle.

**FIGURE 2:**
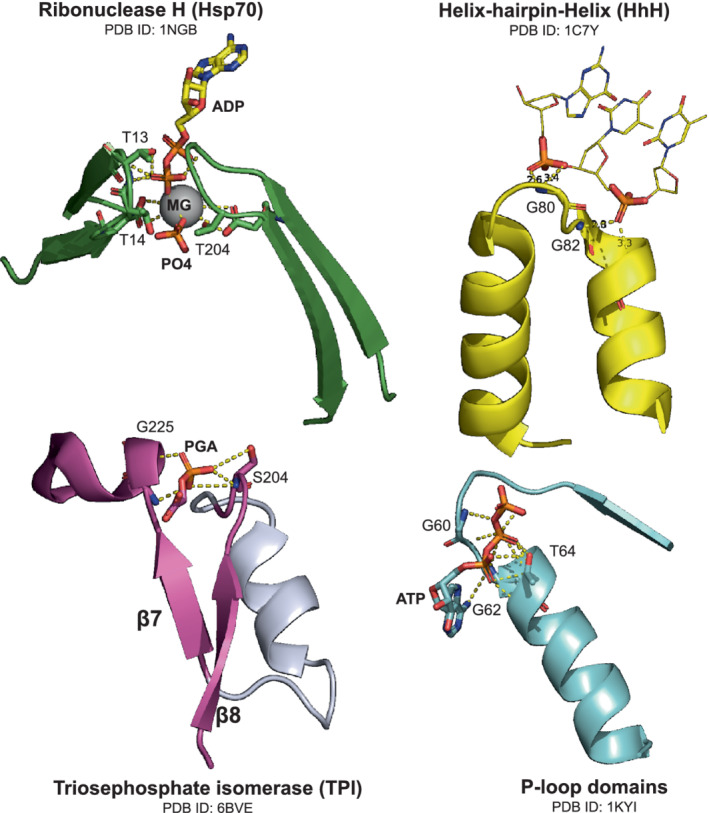
Phosphate binding in the primordial protein world. Shown are various phosphate binding elements, which appear in protein superfamilies that emerged prior to the LUCA. These elements have key characteristics that coincide with early emergence.^7^ First, phosphate binding is realized within a short segment comprising 3–5 residues that provides a “nest” of hydrogen bonds. The primordial binding segments reside mostly at the N‐termini of an ɑ‐helix, as observed in the HhH, Rossmann, and P‐loop NTPase lineages, as well as in the aldolase superfamily to which TPI belongs. Second, phosphate binding is preferentially mediated by abiotic amino acids, foremost by glycine and serine/threonine (the latter often form bidentate hydrogen bonds with the backbone NH and side‐chain OH groups). Note that the phosphate‐binding site of the aldolase superfamily is traditionally assigned to the β‐strands (β7, β8) of the TIM barrel; however, the N‐terminus of a short helix is a critical part.

Evidence supporting the idea that phosphate binding was acquired early is also found in the phosphate‐binding motifs of the earliest enzyme lineages, including those of TPI's lineage. These motifs possess characteristics suggesting that they emerged in the context of the primordial protein world (Figure 2). Overall, it appears that phosphate binding is one of the earliest, if not the earliest, protein function—a function that was exhibited by polypeptides in a primordial world that was based on nucleic acids, amino acids and short peptides, that is, the “peptide‐polynucleotide world”.^8^, ^33^ It is conceivable that these short peptides emerged following an “RNA world” in which both catalysis and genetic information storage were carried out by RNA molecules called ribozymes. In this primordial setting, such ribozymes might have catalyzed the polymerization of amino acids into short peptides,^34^ and phosphate binding by these peptides may have emerged in coordination with these ribozymes. As suggested by Albery and Knowles, an early emergence of enzymes whose substrates contain a phosphate moiety, or even of phospho‐ligand binding peptides, would in turn lead to the enrichment of metabolites that contain a phosphate handle because these would be readily accepted by these preexisting enzymes or peptides (see also recruitment by catalytic promiscuity; Figure 1b). Such “rich get richer” phenomena are manifested in a power law distribution of connectivity in natural metabolic networks.^35^

It should be noted that, even if phosphate served as a “mere handle” in an ancestral enzyme, it may today play some role in the catalytic cycle. During evolution, an enzyme interacting with a non‐reactive phosphate moiety (or any other substrate handle) could have evolved from binding the handle uniformly—that is with equal affinity towards all bound states—towards establishing specific interactions with the transition state. It is well documented for extant TPI and some other phosphate‐binding enzymes that interaction with the phosphate drives protein conformational changes leading to the closed active site exhibiting full transition state complementarity and the reacting moieties being well juxtaposed for catalysis.^15^, ^16^, ^17^

## 3 RESULTS

### 3.1 Can a uniform binder accelerate a reaction?

What advantage might a protein that binds a substrate “handle,” be it phosphate or another moiety, provide? That is, assuming that the phosphate group does not take part in the reaction and that this protein is incapable of selectively stabilizing the transition state. We consider a simple case of a primordial reaction where product formation is irreversible and the rate is determined by the barrier to a single transition state. The height of this barrier is the activation energy ΔG^‡^—the difference in energy between the transition state and the substrate.

**FIGURE 3:**
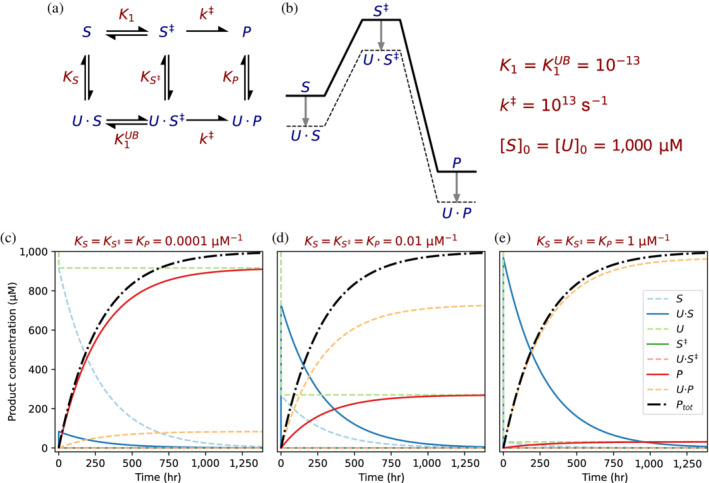
Uniform binding does not accelerate the rate of an uncatalyzed reaction. (a) Substrate S is spontaneously converted to P via a single transition state S^‡^, quantified by the equilibrium constant *K*_1_. The rate of product formation from S^‡^ is given by *k* ^‡^ which is directly proportional to the frequency of the vibrational mode responsible for converting the activated complex to P. A uniform binder U (e.g., a peptide) binds to S, S^‡^, and P with equilibrium constants *K*_*S*_ = *K*_*S*‡_ = *K*_*P*_ (since uniform binding entails equal equilibrium binding constants). (b) Free energy profile for the system shown in. Since all three states are equally stabilized by binding to U, the activation energies of both the bound and unbound reactions are equal (*K*_1_ *= K*_1_^*UB*^). (c–e) Simulation of the model assuming the binding constants are (c) *K*_*S*_ = *K*_*S*‡_ = *K*_*P*_ = 10^−4^ μM^−1^, (d) 10^−2^ μM^−1^, or (e) 1 μM^−1^. In all three cases, the total amount of product (*P*_*tot*_ *= P + UP*, dot‐dashed black line) is the same, regardless of the strength of binding by U.

According to transition‐state theory^2^ the reaction rate is proportional to $e^{-\Delta G^{‡}/\mathrm{RT}}$. Any protein or peptide (U) that uniformly binds the substrate (S), the transition state (S^‡^), and the product (P), is not able to accelerate the reaction beyond the uncatalyzed rate because the activation energy is unchanged (Figure 3b). One way to understand this is by thinking about the uncatalyzed reaction as a decay. Each substrate molecule, whether bound or unbound, has a half‐life *t*_1/2_ and, therefore, the initial rate of product formation will be $\left[S\right]\cdot \ln\left(2\right)/t_{1/2}$ irrespective of the fraction bound to U. In the simulated example (Figure 3), we demonstrate that although the proportions of bound (U·P) versus unbound (P) product greatly depend on the strength of binding, the total amount of product formed (P_*tot*_ = P + U·P) is indeed invariable to it.

The key difference between this analysis and that of Reference 1 is that Albery and Knowles compared an early enzyme to a small molecule catalyst (acetate, in the case of TPI) rather than an uncatalyzed reaction. Indeed, uniform binding can accelerate catalyzed reactions, that is, reactions where Δ*G* ^‡^ is reduced by the presence of a catalyst, by facilitating the association between the substrate and the catalyst. However, it can provide rate acceleration only to a certain extent and becomes counterproductive when binding is too strong.^1^ Uniform binding, furthermore, cannot produce rate acceleration in the unimolecular uncatalyzed case we describe here since the conversion of substrate to product is inherently the rate‐determining step and cannot be accelerated by uniform binding. Of course, it is near certain that prebiotic small molecule catalysts like acetate were present,^36^, ^37^ and therefore, that catalysis might have preceded binding in some cases. However, the new understanding that some enzyme families evolved “binding‐first” (Figure 1) along with his exploration of the limitations of non‐catalytic uniform binders (Figure 3) caused Danny and subsequently us, to wonder: Can uniform binding improve catalysis without providing rate acceleration?

It is important to note that the above analysis applies only to unimolecular reactions. A catalyst might also promote a bimolecular reaction (e.g., S_1_ + S_2_ → P) by binding both substrates. However, the act of bringing together two substrates constitutes an “entropic trap” that lowers the activation energy as compared to the solution reaction. From a mathematical standpoint, bimolecular reactions are quite similar to the case examined by Albery and Knowles wherein uniform binding can improve a proto‐enzyme that already exhibits some degree of rate acceleration by improving substrate binding at the expense of product release.^1^

### 3.2 A binder can divert the reaction's outcome even without rate acceleration

Rate acceleration is the hallmark of enzymes. However, it is often forgotten that the role of an enzyme is not only a matter of making a reaction happen faster. In most biochemical reactions, the impact of an enzyme is manifested in the reaction's product being different from that of the uncatalyzed reaction. Consider, for example, a mixture of dNTPs incubated in two tubes, one containing buffer only and the other buffer plus a polymerase. In the absence of a polymerase, gradual hydrolysis to dNDPs, and then to dNMPs, will take place, resulting eventually in nucleosides plus phosphate. If a polymerase is present, DNA will be formed. Given enough time, DNA will also hydrolyze to give nucleosides and phosphate, which is the thermodynamically favored state regardless of whether the starting point is dNTPs or DNA. However, the hydrolysis of DNA is extremely slow—many orders of magnitude slower than that of nucleotides.^38^ Thus, DNA serves as a kinetic trap. In the intermediate time scale, in effect, for millions of years, the tube containing the enzymatic reaction will contain DNA even though the polymerase is long gone. The tube containing the uncatalyzed reaction will not contain DNA at any time. Hence, diverting reactions away from favorable products and into quasi‐stable “kinetic traps” is critical to life and seen throughout metabolism.

We now show that a binder can divert a reaction away from its favored outcome, provided that the desired product is kinetically trapped. This effect is contingent upon the binder reaching a concentration that matches or exceeds that of the substrate.

**FIGURE 4:**
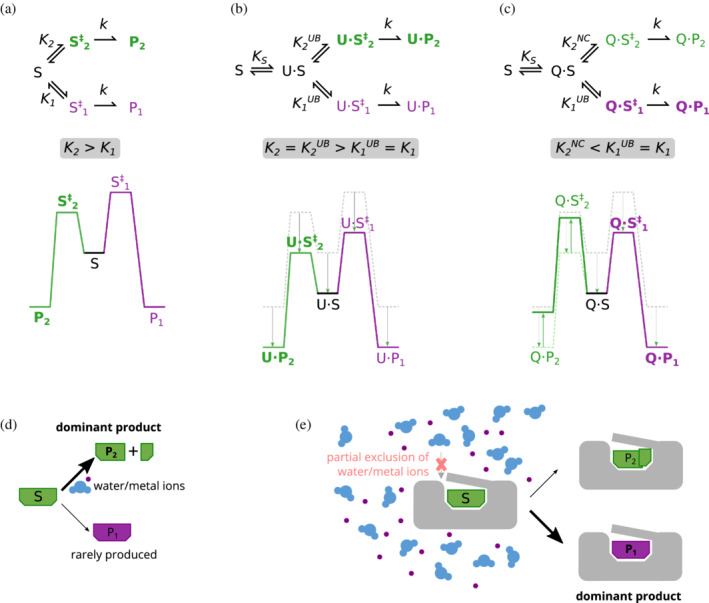
Diversion of a reaction's outcome by uniform binding and negative catalysis. (a) The substrate, S, can react via two different trajectories to give two alternative products, either P_1_ or P_2_. As depicted in a free energy diagram, the dominance of P_2_ in solution is due to a lower transition energy which translates to faster kinetics (*K*_2_ *> K*_1_). (b) The same reaction, mediated via a uniform binder. The free energies of the corresponding non‐bound species, S, S^‡^_1/2_, and P_1/2_, are shown as dashed gray lines, and the outcome of uniform stabilization (an equal reduction in their free energies) as gray arrows. Uniform binding dictates that the transition energy (from U·S to U·S^‡^_1/2_) remains the same, and therefore that *K*_2_^*UB*^ *= K*_2_ and *K*_1_^*UB*^ *= K*_1_. Consequently, *K*_2_^*UB*^ *> K*_1_^*UB*^ and P_2_ remains the dominant product. (c) Q is similar to U with regard to the energy profile of the S → P_1_ reaction, but performs negative catalysis with regards to the S → P_2_ reaction. This is depicted as a relative destabilization of Q·S^‡^_2_ and Q·P_2_ compared to U·S^‡^_2_ and U·P_2_ (as shown by the light‐green arrows). Consequently, the barrier to P_2_ is higher than for P_1_, making P_1_ the dominant product (*K*_1_^*UB*^ *> K*_2_^*NC*^). (d) Diagram of a setting where negative catalysis is desirable. In solution, the dominant reaction S → P_2_ might require water or metal ions. The desirable reaction S → P_1_ is much slower and so P_1_ is rarely produced. (e) Partial exclusion of solvent is a possible mechanism for negative catalysis. Binding sequesters S, but has the side effect of excluding solvent components that promote the S → P_2_ reaction, that is, slowing the undesirable reaction by raising the Q·S^‡^_2_ barrier (negative catalysis, panel c). If S → P_1_ is unaffected, P_1_ becomes the dominant product.

Consider a substrate that can react in two different ways to give two alternative products, P_1_ and P_2_ (Figure 4a). In solution, P_2_ is kinetically favored as it has a lower activation energy (*K*_1_ *< K*_2_). Now consider the same reaction proceeding via a uniform binder U (Figure 4b). This binder uniformly reduces the free energy of all bound states, namely the substrate‐binder complex (U·S), the complexes with the transition states (U·S_1/2_^‡^), and the complexes with the products (U·P_1/2_). Therefore, the rates of formation of P_1_ and P_2_ do not change and neither does the reaction outcome. Alternatively, consider a scenario where the transition state leading to P_2_ is not stabilized to the same extent as for P_1_ (Figure 4c; uniform binding for S to P_1_ and negative catalysis for S to P_2_). We denote such a binder Q to distinguish it from a standard uniform binder. As argued above, the rate constant for the conversion of Q·S into Q·P_1_ remains unchanged, namely *K*_1_ *= K*_1_^*UB*^. Accordingly, the actual rate of formation of Q·P_1_ will, at best, match the rate in solution. Furthermore, P_1_ would be mostly bound to Q, rather than unbound in solution. However, since Q does not stabilize Q·S_2_^‡^ to the same extent as Q·S_1_^‡^, the barrier for conversion to P_2_ may become higher than the barrier to P_1_ (*K*_1_^*UB*^ *> K*_2_^*NC*^) and, consequently, Q·P_1_ will be formed at a faster rate than Q·P_2_. Thus, Q can divert the reaction by allowing a kinetically disfavored product to become the dominant outcome.

In some enzymes, negative catalysis, that is, suppression of undesirable reactions that readily occur in solution, is as critical as positive catalysis of the desired reaction.^39^, ^40^ Curiously, negative catalysis is also relevant for TPI. The interconversion of dihydroxyacetone phosphate and glyceraldehyde‐3‐phosphate proceeds via an enediol intermediate. In solution, this intermediate would rapidly give methylglyoxal (with the phosphate serving as a leaving group). However, in the enzyme's active site, methylglyoxal is produced at a frequency of ~10^−6^ compared to glyceraldehyde‐3‐phosphate.^41^ It is the binding configuration of the phosphate that likely prevents elimination of the enzyme‐bound enediol.^16^, ^40^, ^42^ Interestingly, negative catalysis can be mediated by residues distal to the active site as well. For example, the mitochondrial enoyl‐thioester reductase (Etr1p), which reduces enoyl‐CoA by NADPH, uses a remote threonine to destabilize only the transition state for the formation of a dead‐end product, a C4‐adduct of the enolate intermediate and NADP^+^.^43^ This threonine appears to stabilize an arrangement of the active site (including ordered waters) that suppresses formation of the unwanted product by a factor of ≈10^6.^

A different outcome can be achieved via negative catalysis because Q “hijacks” the substrate and in a way compartmentalizes it, provided that the concentration of Q is commensurate with the substrate concentration. In doing so, it can make the dominant solution reaction (S to P_2_) less favorable, for example via the exclusion of bulk water (like the slowing of dNTP hydrolysis in the polymerase example at the beginning of this section, Figure 4d,e). Similarly, charge dipoles, or hydrogen bond donors and acceptors, may destabilize transition states as much as they stabilize them. The point is not that Q differentially stabilizes the transition state to the desired product P_1_ relative to the bound substrate, as this would have been a case of positive catalysis. The key element is rather a relative destabilization of the transition state leading to the undesirable product P_2_ (Figure 4b,c).

### 3.3 The merit of “negative catalysis” with neither rate acceleration nor product release

**FIGURE 5:**
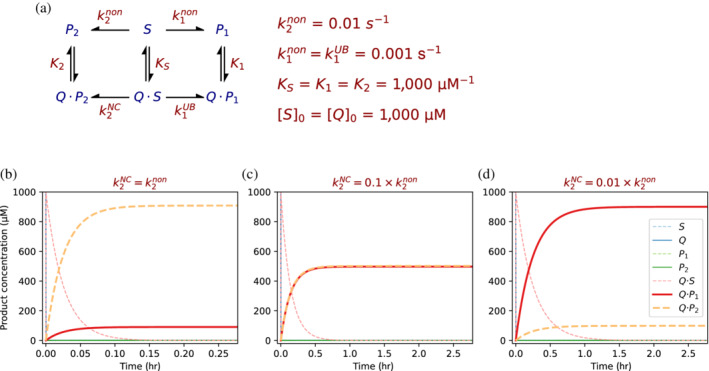
Negative catalysis of a reaction leading to an undesirable product. (a) The model assumes that a substrate can react to give either the product P_1_, which is the desired but disfavored product, or P_2_ which is favored in solution (*k*_2_^*non*^ = 10 *k*_1_^*non*^). (b) In the presence of a uniform binder that does not alter the rate of formation of P_1_ nor of P_2_, (corresponding to Figure 4b, where Q is actually U in this case), the P_1_:P_2_ ratio remains 1:10, as in solution. Note, however, that the outcomes are the corresponding product complexes Q·P_1/2_ while the concentrations of the unbound products P_1/2_ are effectively nil. (c) The product ratio changes if the binder stabilizes the transition state leading to P_2_ to a lesser extent than the transition state leading to P_1_, here by 1.4 kcal mol^−1^. This results in a 10‐fold reduction in the rate of S‐to‐P_2_ conversion when the substrate is bound to the binder (*k*_2_^*NC*^ = 10^−3^ s^−1^ compared to *k*_2_^*non*^ = 10^−2^ s^−1^). The outcome under these conditions is P_1_:P_2_ ≈ 1. (d) This time, corresponding to Figure 4c, a 2.8 kcal mol^−1^ differential destabilization of S_2_^‡^ leads to a 100‐fold rate reduction for S‐to‐P_2_. The result is P_1_ now predominating over P_2_, at a ratio of P_1_:P_2_ ≈ 10:1.

To simulate how a binder (Q) may redirect the reaction's outcome, let us first consider a scheme where, in solution, P_2_ is formed at a rate that is 10 times higher than P_1_. Once the solution reaction comes to completion, these products would be present at a 1:10 ratio. As argued above, the presence of a uniform binder (U) would not alter this ratio. However, if the rate of conversion of the bound substrate to P_2_ decreases, the fraction of P_1_ will increase (Figure 5). Note that the rate of P_1_ formation cannot increase because Q does not accelerate this reaction (i.e., *k*_1_^*non*^ *= k*_1_^*UB*^). The only requirement is that the Q‐mediated conversion rate of S to P_2_ is sufficiently reduced. Another prerequisite for the dominance of P_1_ is that the Q is present at concentrations that are at least as high as the substrate ([Q]_0_ ≥ [S]_0_). This is because the key to the binder's effect is the rapid sequestration of the substrate, thus preventing the competing solution reaction.

The effect of Q in shifting the reaction products would increase with greater uniform stabilization of S, S_1_^‡^, and P_1_, but only if this stabilization does not apply equally to S_2_^‡^ and P_2_. In other words, the effectiveness of negative catalysis by Q depends on the lesser stabilization of the transition state leading to P_2_ versus the one leading to P_1_. The lower the stabilization of Q·S_2_^‡^ (represented in this model via a lower *k*_2_^*NC*^) the greater the steady state P_1_:P_2_ ratio (Figure 5b–d).

The binder's concentration and the degree of negative catalysis are the key factors determining the steady‐state P_1_:P_2_ ratio in this model (Figure S1). Strikingly, a hundred‐fold inhibition of P_2_ formation (i.e., *k*_2_^*NC*^ = 0.01 × *k*_2_^*non*^) leads to the dominance of P_1_ over P_2_ even when starting Q and S concentrations are equal ([Q]_0_ = [S]_0_, Figure 5d). The snag is that P_1_ accumulates in the bound state, that is, as Q·P_1_ (Figure 5b–d). This outcome is a simple consequence of the definition of uniform binding (i.e., equal binding of S and P_1_ by Q) and the need to sequester S by binding in order to prevent the dominant solution reaction (i.e., leading to P_2_). Thus, as elaborated below, such binders would be most effective in pathways where a subsequent favorable reaction pulls the equilibrium in favor of product release.

### 3.4 Are the advantages of negative catalysis evolutionarily meaningful?

The need for a higher protein concentration than substrate concentration is a shackling constraint ([Q]_0_ ≥ [S]_0_). What Albery and Knowles termed “catalysis of elementary steps”,^1^ namely binding the transition state more tightly than the substrate, is the only way of alleviating this constraint. How feasible is it, then, for a binder, which cannot accelerate the reaction rate, to provide a selectable physiological advantage through negative catalysis?

The above question is not limited to the herein presented binders—it applies to any protein that mediates a potentially beneficial reaction with a very low rate, and whether or not such a protein can serve as an evolutionary starting point. Mutations can enhance very weak activities. However, for these mutations to be fixed, the target protein must confer some initial advantage such that it is “seen” by selection. In some cases promiscuous activities are high, with *k*_*cat*_/*K*_*M*_ values in the order of 10^3^ s^−1^ M^−1^, or even higher (recall that the average *k*_*cat*_/*K*_*M*_ for all known enzymes is 10^5^ s^−1^ M^−1^).^44^ Direct evidence that a promiscuous activity at this level provides a growth advantage has been obtained.^45^ However, most reported promiscuous activities are far lower, and *k*_*cat*_/*K*_*M*_ values under 1 s^−1^ M^−1^ are not uncommon.

Not much is known regarding the activity thresholds that are “visible” to selection, but these might be surprisingly low. We, for example, reconstructed the trajectory that led from an ancestor to a catalytically efficient stereoselective isomerase.^46^ While the fully reconstructed ancestor showed no catalytic activity at all, the initial mutations did give rise to a very low isomerase activity that was detectable only when the protein concentration was higher than the substrate. Furthermore, these early intermediates generated a single stereoisomer, where the uncatalyzed reaction yielded a racemic product. This would have made them beneficial and selectable in the ancestral land plant in which this enzyme emerged, provided that the protein concentration was high enough.

Note, however, that protein concentrations need not be high per se, but only high compared to the substrate. The substrate of an emerging enzyme is often in itself the product of a coincidental, promiscuous reaction (enzymatic or even non‐enzymatic)^35^ and would likely be present only at low concentrations. Further, high local protein concentrations are feasible (via clustering, membrane association, etc.), and enzymes that act stoichiometrically are known. An example is the dirigent proteins that direct the regio‐ and stereo‐specificity of plant natural products.^47^ Achieving enantiomer specificity may well be a case of negative catalysis, namely of destabilizing the transition state leading to the undesirable enantiomer.

### 3.5 The key role of kinetic trapping of products

**FIGURE 6:**
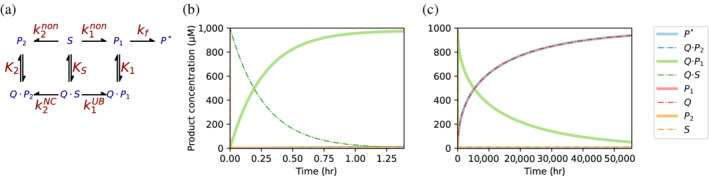
The effect of coupled reactions and kinetic trapping of the product. (a) The reaction scheme used to simulate coupled reactions. The scheme is the same as in Figure 5, with the uniform binding equilibrium constants *K*_*S*_ = *K*_1_ = *K*_2_ = 10^4^ μM^−1^, and a 1,000‐fold slower rate of conversion to P_2_ for the bound substrate compared to unbound, namely *k*_2_^*NC*^ = 10^−5^ s^−1^ versus *k*_2_^*non*^ = 10^−2^ s^−1^. As before, for P_1_ there is no difference between the rates: *k*_1_^*non*^ = *k*_1_^*UB*^ = 10^−3^/s. The binder's product, P_1_, further reacts irreversibly to give the final product P*, with a forward rate constant, *k*_*f*_, of 0.1 s^−1^. (b) The concentrations of the different species as a function of time during the first 100 min, during which almost all of the substrate is converted to P_1_ and is bound to Q. (c) The concentrations of the different species in longer time‐scales, where P* becomes the dominant species and Q returns to its unbound state.

Binders that are poor catalysts or non‐catalysts can provide a distinct advantage in the context of a reaction sequence. As seen in Figure 4, a binder can prevent the formation of the undesirable product (P_2_). Unfortunately the desired product (P_1_) is almost entirely trapped in the bound state (Q·P_1_). However, if P_1_ reacts further, via a spontaneous reaction or an enzymatic one, its conversion to the next product (P*) ensures that more P_1_ is released. After release, Q can participate in another round of S → P_1_ → P* reactions. The rate of the coupled reaction (P → P*) can be slow, even in the same range as the conversion of the protein‐bound substrate to product. Nevertheless, for P* to accumulate, P_1_ → P* must be a highly exergonic reaction (Figure 6).

Coupling of reactions is a hallmark of living systems. In the context of coupled reaction sequences, poor catalysts can be beneficial. As seen in Figure 6, the presence of Q eventually results in the desired product (P*), in its unbound form, dominating over the product favored in solution (P_2_). Given no rate acceleration, the rates of such processes will remain as slow as they were before, but their outcome is ensured. Note that in this coupled mode, dominance of the desired product (P*) is still only achieved when [Q]_0_ ≥ [S]_0_; stoichiometric amounts of the binder remain a prerequisite in order to sequester free S and redirect the spontaneous reaction.

## 4 DISCUSSION

### 4.1 From a substrate‐handle binder to a bona fide enzyme

A trajectory for the emergence of a new enzyme may begin with a protein binding a substrate via a “handle” such as a phosphate moiety. A favorable outcome is obtained because substrate‐handle binding out‐competes the uncatalyzed reaction and hence diverts the reaction to an alternate product—for example, by exclusion of bulk water (Figure 4e) and/or by some promiscuous interactions with protein residues. As noted above, this mode of negative catalysis may involve transition state stabilization, but stabilization can be uniform, namely with no discrimination in favor of the transition state compared to S and P. At later stages of enzyme evolution, mutations might arise that preferentially stabilize the transition state over S (i.e., *K*_*S*‡_ > *K*_*S*_ in Figure 3). This provides rate acceleration also at sub‐stoichiometric protein concentrations ([E]_0_ < [S]_0_) by increasing *k*_*cat*_ and accelerating turnover of the enzyme. Differential binding of substrate and product would also be beneficial to evolve in a uniform binder.

The above emergence scenario may apply to various stages of evolution and may begin with the recruitment of an existing enzyme, or even a non‐catalytic protein, via promiscuous substrate‐handle binding (see Figure 1d). However, this scenario is of particular relevance to the early stages of evolution. At the dawn of life, simple organic or even inorganic molecules (e.g., crystalline minerals, as in Maynard‐Smith's clay model^48^) could have acted as substrate binders, especially because, as shown above, with this mode of action, even very low affinities can exert an effect (Figure 5). Of course, such prebiotic binders could not themselves evolve to be proficient catalysts, but they may have played a role in bringing chemistry into an appropriate timescale for biology. At later stages, such as the presumed “peptide‐polynucleotide world,”^8^ short peptides that bound nucleotides and nucleic acids would have played a key role. As shown here, these peptides can act, with no rate acceleration, to redirect the reaction's outcome (e.g., polymerization reactions of bound RNAs).

While we conceptualized this process of catalysis emergence for peptide‐based protoenzymes in a “peptide‐polynucleotide world,”^8^ such a scheme is also suitable for other hypothetical conditions of prebiotic chemistry. Our proposed scenario for the emergence of catalysis may have also played a role in an “RNA world,” where ribozymes might have first emerged as substrate binders that evolved to divert an uncatalyzed reaction toward a preferred product.

Peptides that bind phospho‐ligands have been described, including peptides of prehistorical relevance. Most notable are peptides derived from the P‐loop NTPases and Rossmann enzymes—two key enzyme lineages that originated well before LUCA.^20^ So‐called P‐loop prototypes have been described that contain the phosphate‐binding loop of P‐loop NTPases (also known as the Walker A motif).^33^ This eight‐residue long loop is embedded within its original structural context (β‐strand‐P‐loop‐α‐helix), yet lacks the rest of the three‐layer sandwich domain that comprises at least five strands and four helices. These P‐loop prototypes bind a range of phospho‐ligands, including inorganic phosphoanhydrides (tri‐ or poly‐phosphates), ribonucleotides such as ATP or GTP, and also DNA and RNA. They show some selectivity, for example, the differential binding of ssDNA over dsDNA, but are unlikely to exhibit differential transition‐state stabilization for phosphoryl transfer (unlike the contemporary P‐loop NTPases). Nonetheless, differential binding of ssDNA and dsDNA in their ground states enables the P‐loop prototypes to induce helicase‐like strand‐separation and also to accelerate the rate of strand exchange (a recombinase‐like activity).^33^ Their action is stoichiometric, demanding peptide concentrations that are higher than those of their DNA substrates. The bound ssDNA can, however, be released by competing phospho‐ligands (ATP or polyphosphate). High protein concentration is unavoidable for binders that do not accelerate the reaction rate and other proto‐enzymes that exhibit very low catalytic efficiency. However, high peptide/protein concentrations could have been achieved in primordial life forms, for example, in coacervates (liquid‐liquid phase separation) that readily form upon mixing of certain peptides with RNA or even with small molecules such as ATP.^49^

In the context of the primordial world, peptides that lacked the catalytic capabilities of modern enzymes would have needed to provide an alternative evolutionary benefit in order to be selected. Due to their relative simplicity, mechanisms based on uniform binding (e.g., of a phosphate‐handle) were probably the first to evolve as they do not require discriminating between the substrate, product, and transition state. These binders could have provided catalysis of chemical reactions by facilitating the binding of a substrate to a pre‐existing catalyst, or, in the case of a bimolecular reaction, by acting as an entropic trap. Here, we have shown that although there are cases when uniform binding by peptides is unable to improve the catalytic rate (such as uncatalyzed, unimolecular reactions), such binders may still provide a fitness advantage by channeling metabolism towards desired products—with the help of negative catalysis.

## 5 MATERIALS AND METHODS

### 5.1 Model‐based simulations

We simulated the trajectories of substrates, products, and binders using models based on ordinary differential equation systems. The Jupyter notebook that we used to run the simulations and generate Figures 3, 5, and 6, and S1 is stored in a dedicated GitLab repository:https://gitlab.com/milo-lab-public/negative-catalysis/-/blob/main/generate_figures.ipynb. An interactive version of Figure 3 where one can adjust the initial concentration of U (as well as the binding coefficient) is also available: https://mybinder.org/v2/gl/milo-lab-public%2Fnegative-catalysis/HEAD?labpath=figure3_interactive.ipynb

## AUTHOR CONTRIBUTIONS

**Elad Noor:** Formal analysis (equal); resources (equal); software (equal); visualization (equal); writing – review and editing (equal). **Avi I. Flamholz:** Formal analysis (equal); resources (equal); software (equal); visualization (equal); writing – review and editing (equal). **Vijay Jayaraman:** Data curation (equal); investigation (supporting); methodology (equal); writing – review and editing (equal). **Brian L. Ross:** Data curation (equal); investigation (supporting); methodology (equal); writing – review and editing (equal). **Yair Cohen:** Investigation (supporting); writing – review and editing (equal). **Wayne M. Patrick:** Conceptualization (supporting); investigation (supporting); project administration (lead); writing – review and editing (equal). **Ita Gruic‐Sovulj:** Conceptualization (supporting); investigation (supporting); supervision (lead); writing – review and editing (equal). **Dan S. Tawfik:** Conceptualization (lead); funding acquisition (lead); investigation (lead); writing – original draft (lead).

## Supporting information


**Appendix S1** Supporting information
