# Supplementary material for: Uniform binding and negative catalysis at the origin of enzymes
Source: Protein Sci. 2022 Jul 14;31(8):e4381. doi: 10.1002/pro.4381 (PMC9281367; doi:10.1002/pro.4381)
Supplement: Supplementary file 1 — Appendix S1 Supporting information [file PRO-31-e4381-s001.docx]

# **Uniform binding and negative catalysis at the origin of enzymes**

Elad Noor^1*✡^, Avi I. Flamholz^2✡^, Vijay Jayaraman^3✡^, Brian L. Ross^4✡^, Yair Cohen^5^, Wayne M. Patrick^6^, Ita Gruic-Sovulj^7*^, Dan S. Tawfik^3^

^1^ Department of Plant and Environmental Sciences, Weizmann Institute of Science, Herzl 234, Rehovot 7610001, Israel

^2^ Division of Biology and Biological Engineering, California Institute of Technology, Pasadena, CA 91125

^3^ Department of Molecular Cell Biology, Weizmann Institute of Science, Rehovot 7610001, Israel

^4^ Department of Biomolecular Sciences, Weizmann Institute of Science, Rehovot 7610001, Israel

^5^ Department of Caltech Environmental Science and Engineering, California Institute of Technology, Pasadena, CA 91125

^6^ School of Biological Sciences, Victoria University of Wellington, Wellington 6012, New Zealand

^7^ Department of Chemistry, Faculty of Science, University of Zagreb, Horvatovac 102a, 10000 Zagreb, Croatia

^✡^ These authors contributed equally

* Corresponding authors

Prof. Ita Gruic-Sovulj

Department of Chemistry, Faculty of Science, University of Zagreb

Horvatovac 102a, 10000 Zagreb, Croatia

e-mail: [gruic@chem.pmf.hr](mailto:gruic@chem.pmf.hr)

Elad Noor

Department of Plant and Environmental Sciences, Weizmann Institute of Science,

Herzl 234, Rehovot 7610001, Israel

e-mail: [elad.noor@weizmann.ac.il](mailto:elad.noor@weizmann.ac.il)

## Supplementary material

### Supplementary Table S1

Table containing a list of LUCA reactions containing a non-reacting phosphate group:

<https://gitlab.com/milo-lab-public/negative-catalysis/-/blob/main/data/Table_S1_non_reactive_phosphate_reactions.csv>

### Supplementary Figure S1


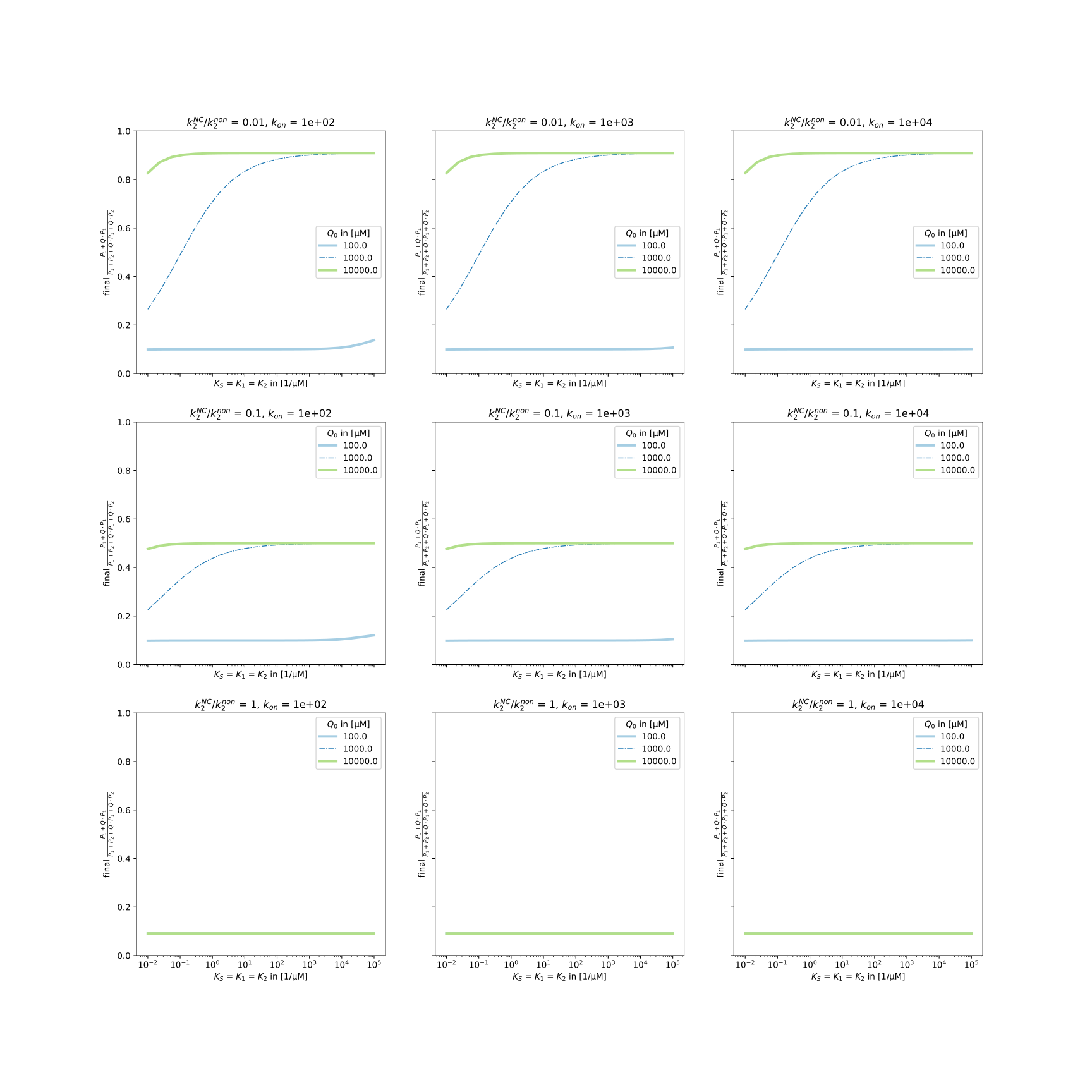


**Supplementary Figure 1:** Sensitivity analysis of the model of uniform binding coupled to negative catalysis presented in Figure 5. Each panel plots the degree of uniform binding (*K_S_ = K_1_ = K_2_*) against the relative concentration of the desired product, P_1_, at steady-state (P_1_ + Q·P_1_ / (P_1_ + P_2_ + Q·P_1_ + Q·P_2_)). The degree of negative catalysis (*k_2_^NC^/k_2_^non^*) decreases along the rows, while the on-rate (*k_on_*) associated with Q·S binding increases along the columns. Note that the model enforces *k_on_* / *k_off_ = K_S_.* The initial substrate concentration [S]_0_ was set to 1000 μM throughout, and colored curves denote different starting concentrations [Q]_0_ of the binder as indicated in the legend. We see that *k_2_^NC^/k_2_^non^* sets the maximum steady-state P_1_ level, while [Q]_0_ and *K_S_* determine whether this maximum is realized.
